# HIV Status Disclosure Among Perinatally Infected Adolescents Living With HIV (ALHIV): Perspectives of Primary Caregivers, Healthcare Providers, and ALHIV in Mumbai, India

**DOI:** 10.7759/cureus.108703

**Published:** 2026-05-12

**Authors:** Shrikala Acharya, Mrinalini Das, Sreevidya P. A., Bhawana Shinde, Sushil Nikam, Vidula Patil, Jekin Choubisa, Anjali Sharma, Ram H Malkani, Maninder S Setia

**Affiliations:** 1 Community Medicine, Lokmanya Tilak Municipal Medical College and General Hospital, Mumbai, IND; 2 Medicine, Joint Effort for Elimination of Tuberculosis (JEET), Foundation for Innovative New Diagnostics (FIND) India, New Delhi, IND; 3 Public Health, University of Washington-International Training and Education Center for Health, New Delhi, New Delhi, IND; 4 Public Health, Mumbai Districts AIDS Control Society, Mumbai, IND; 5 Clinical Research, Dr. Skin Pimples Pvt. Ltd., Mumbai, IND; 6 Dermatology, Dr. Skin Pimples Pvt. Ltd., Mumbai, IND; 7 Epidemiology, Mahatma Gandhi Mission Institute of Health Sciences, Mumbai, IND

**Keywords:** adolescents, caregivers, children, disclosure of hiv status, health care providers, qualitative study

## Abstract

Background

Advances in pediatric HIV care have increased survival beyond adolescence, but disclosure of HIV status remains challenging. Many adolescents are unaware of their status and face psychosocial difficulties related to lifelong treatment adherence. We conducted a qualitative study to explore the perspectives of all three stakeholders (children and adolescents living with HIV (C/ALHIV), primary caregivers, and healthcare providers (HCPs)) and understand the challenges and nuances surrounding the process of disclosing HIV status to children and adolescents with HIV. We also aimed to identify the enablers and barriers to disclosure.

Methods

A qualitative study was conducted involving 24 interviews and eight focus group discussions (FGDs) with C/ALHIV (HIV status was disclosed), caregivers of C/ALHIV (HIV status was not disclosed to children and adolescents), and HCPs from seven antiretroviral therapy (ART) centres in Mumbai, including a pediatric ART centre. The FGDs and interviews were conducted in Hindi, Marathi, or English (according to the language preference). These recorded interviews were transcribed and translated into English for analysis. Thematic network analysis was used to identify key themes related to the disclosure process.

Results

Disclosure was perceived as complex and emotionally challenging. Key themes included apprehensions of caregivers and HCPs about disclosure-related emotional distress, C/ALHIV’s desire for accurate information, dilemmas regarding timing and responsibility for disclosure, experiences of incomplete disclosure, adverse psychological effects such as depression and ART non-adherence, and a lack of structured follow-up counselling after disclosure. C/ALHIV reported ongoing anxiety related to social stigma and accidental disclosure risks. Enablers in the disclosure process included individualized counselling, while barriers involved insufficient provider knowledge, inadequate counselling protocols, and poor documentation.

Conclusion

This study highlights the multifaceted challenges of HIV status disclosure to C/ALHIV in India. Findings support developing comprehensive, culturally sensitive guidelines emphasizing collaborative disclosure approaches, HCP training, and structured follow-up care to enhance comprehensive care and management of adolescents living with HIV.

## Introduction

HIV continues to be a global public health concern. According to the United Nations International Children’s Emergency Fund (UNICEF), approximately 1.38 million children aged 0-14 years are currently living with HIV, with children under one year of age being most vulnerable to the infection [1]. Additionally, it has also been reported that less than 60% of these are on antiretroviral therapy (ART), even though children under the age of 15 contribute to about 12% of AIDS-related mortality [1].

Children and adolescents are identified as vulnerable groups that may require specialized programmes within the context of the global AIDS response. Substantial advancements in pediatric HIV prevention and treatment, such as the availability of pediatric ART formulations, have changed the epidemic and improved the survival of children who were infected perinatally [2,3]. It is possible that many children will live into adolescence and beyond. However, this may present new challenges, as many of these adolescents may be unaware of their HIV status and must navigate both the biological and psychosocial demands of growing up while adhering to lifelong treatment [4-6]. Disclosure of HIV status to them becomes one of the most significant psychosocial difficulties faced by caregivers and healthcare providers (HCPs) [7].

Determining when and how to disclose HIV status to a child living with HIV represents a central, ethical, and practical dilemma for caregivers [8]. Many caregivers delay telling children about their HIV diagnosis until the child is young. Fear of stigma and of revealing the parents’ status, uncertainty about how to explain the diagnosis, concern about upsetting the child, and the belief that the child is too young to understand are some of the reasons that contribute to this practice [9-12]. Studies also reveal potential risks such as suicidal tendencies and self-harm in children and adolescents living with HIV (C/ALHIV) on disclosure [12]. According to a systematic review by Vreeman et al., the worldwide rate of disclosure amongst children and adolescents in resource-limited settings ranged from 0 to 69.2% [13]. Research has identified various child-specific, caregiver-related, HCP, and sociocultural factors, each unique to different cultural contexts, that influence the disclosure of a child’s HIV status [14-18]. Disclosure of information can be done in two ways: partially, where certain stigmatizing aspects are omitted (such as not mentioning HIV by name), or fully, where all details about HIV are revealed [19,20]. According to WHO guidelines, the disclosure process should begin at the age of six and be completed before the child reaches 12. It recommends that disclosure should occur incrementally and at an appropriate time, considering both the child’s cognitive skills and emotional maturity [21]. However, the implementation of these guidelines varies widely across healthcare settings; in real-world, low-resource environments, they are not always followed or feasible [10,14]. Research suggests that C/ALHIV are often informed of their status much later in life, typically between ages 17 and 18. This delay is frequently driven by cultural beliefs that children are too immature to cope, fears that they will become depressed or lose hope, and concerns regarding social discrimination [14].

According to the latest estimates, India has an estimated 2.56 million people living with HIV (PLHIV); of these, about 57,000 are children under the age of 15 [22]. With the successful implementation of the "Prevention of Parent to Child Transmission (PPTCT)" program and strong investment in HIV treatment, India has witnessed an increased survival of children who acquired HIV perinatally [23]. Disclosure of HIV status to C/ALHIV, however, continues to remain low [24]. Various studies have observed that disclosure promotes positive outcomes such as the development of trust, better adherence, enhanced access to support services, open family communication, and improved long-term health and emotional well-being in children [8,25,26]. Previous Indian studies have reported a disclosure rate varying from 14% to 41% [27-29]. India’s National Guidelines for HIV Care and Treatment (2021) recommend initiating the disclosure process at the age of four to six years and completing full disclosure by 12-14 years [30]. It recommends that parents/caregivers share information with the children. In case of caregivers not being comfortable with the task, common counselling sessions with the HCP, child, and the caregiver are recommended [30]. Although such age-appropriate disclosure is advocated in the national guidelines, its practical implementation at ART centres varies widely.

Understanding disclosure from the perspectives of all three key stakeholders - C/ALHIV, their caregivers, and HCPs - may potentially help us develop culturally appropriate, comprehensive guidelines for disclosure in C/ALHIV.

Aims and objectives

Our primary aim in conducting this qualitative research was to explore the perspectives and experiences regarding the HIV disclosure process for C/ALHIV among the various stakeholders. Through this study, we also tried to understand the enablers and barriers in implementing disclosure guidelines.

## Materials and methods

Study design

This is a qualitative study using focus group discussions (FGDs) and in-depth interviews with C/ALHIV (HIV status disclosed to them), caregivers of children and adolescents (HIV status not disclosed to them), and HCPs.

Study setting and population

The study was conducted in Mumbai, Maharashtra, India. At an estimated 399,000 cases, Maharashtra has the highest number of PLHIV in India, and Mumbai, the capital city of Maharashtra, is considered to be a high burden district [22]. Mumbai District AIDS Control Society (MDACS) is the apex body that implements the National AIDS Control Program and provides HIV care, support, and treatment services in the city through 20 antiretroviral treatment (ART) centres. The pediatric ART centre at a tertiary care institute caters to 60% of all C/ALHIV enrolled for care. The other six ART centres with a case load of more than 30 C/ALHIV were selected for wider representation.

Study population and participants

C/ALHIV are enrolled for care at ART centers across Mumbai according to their place of residence and their parents’ designated ART center. One is an exclusive pediatric ART centre, and most of the children (including institutionalized orphans) are registered for care at this pediatric ART centre in the city. Additionally, HCPs and guardians of children on ART (primary caregivers) were enrolled from this pediatric ART centre and six other ART centres in the city. Thus, all the study participants were recruited from seven ART centres. A total of 24 in-depth interviews and eight FGDs were conducted in the present qualitative study. We used purposive sampling to select the participants for these FGDs and in-depth interviews [31]. C/ALHIV who were aware of their HIV status for at least six months were considered as HIV disclosed individuals, and C/ALHIV who were not aware of their HIV status or if HIV status was not disclosed to them were considered as HIV undisclosed individuals. A minimum period of six months since disclosure was selected to minimize the influence of immediate emotional or impulsive reactions following disclosure, while still ensuring that participants could reliably recall their experiences without recall bias.

The inclusion criteria for C/ALHIV FGDs and interviews were C/ALHIV, aware of their HIV status for at least six months, and enrolled for care at the ART centre for at least more than one year at least, and C/ALHIV who were not aware of their HIV status but enrolled for care at these centres for at least one year. The inclusion criteria for primary caregivers of C/ALHIV included mothers, fathers, and any family member staying with the beneficiaries and taking care of their daily needs. The HCPs included in the study are the medical officers, counsellors, outreach workers, and the care coordinators providing clinical care, counselling, or peer support to C/ALHIV registered for treatment at these ART centres. Participants for the interviews and FGDs (C/ALHIV, primary caregivers) were purposively selected through referrals from counsellors. HCPs were purposively selected for in-depth interviews from seven ART centres. The details of the interview and FGD participants are given in Table 1. The two FGDs, each with boys and girls, ensured representation of the disclosure-related experience of both genders.

**Table 1:** Participants included in focus group discussions (FGDs) and in-depth interviews, Mumbai, India ART: antiretroviral therapy, C/ALHIV: children and adolescents living with HIV

Participants	Interview/FGD	Number and site
C/ALHIV (14-19 years)		
Boys - HIV Status disclosed	FGD	2 (1 - Pediatric ART Centre, 1 - Other Centre)
Girls - HIV Status disclosed	FGD	2 (1 - Pediatric ART Centre, 1 - Other Centre)
Boys - HIV Status disclosed	Interview	4 (2 - Pediatric ART Centre, 2 - Other Centre)
Girls - HIV Status disclosed	Interview	4 (2 - Pediatric ART Centre, 2 - Other Centre)
Caregivers of C/ALHIV		
C/ALHIV (<10 years)	FGD	2 (1 - Pediatric ART Centre, 1 - Other Centre)
C/ALHIV (10-19 years)	FGD	2 (1 - Pediatric ART Centre, 1 - Other Centre)
C/ALHIV (<10 years)	Interview	4 (2 - Pediatric ART Centre, 2 - Other Centre)
C/ALHIV (10-19 years)	Interview	4 (2 - Pediatric ART Centre, 2 - Other Centre)
Healthcare Providers		
Medical Officers	Interview	2 (1 - Pediatric ART Centre, 1 - Other Centre)
Counsellors	Interview	2 (1 - Pediatric ART Centre, 1 - Other Centre)
Care Co-ordinators	Interview	2 (1 - Pediatric ART Centre, 1 - Other Centre)
Outreach Workers	Interview	2 (1 - Pediatric ART Centre, 1 - Other Centre)

The sample size for the FDGs and in-depth interviews was decided a priori to ensure a selection of various types of participants based on the numbers of C/ALHIV, caregivers, and HCPs in the ART centres. A preliminary review of the recorded data of each FGD and interview was conducted to identify initial themes. We probed some of these themes in the subsequent FGD and interview. Though we did not have a formal analysis for thematic saturation [32,33], continuous review of the recorded FDGs and interviews was done to ensure no additional points were being provided after the last FGD or interview.

Data collection

FGDs and in-depth interviews were conducted by a trained qualitative researcher, using open-ended questions in the guides in English (for HCPs) and in Hindi or Marathi (for other participants). We used separate interview guides for each population type. Some topics in the guide for C/ALHIV who knew about their status were knowledge about HIV and treatment, their feelings, and response to the infection, how does it affect their lives including leisure activities, adherence to ART and missed medications, support from healthcare workers and clinical team, suggestions to improve care and support services, time and method of disclosure of HIV status, reaction to disclosure, issues after disclosure, and the information accessed related to HIV by them. Some topics in the guide for caregivers were their child’s reaction to illness and treatment, what motivates the child to take medications, and details about missed medications, side effects of medications, and the child's response, information to the child, support by the ART centre, and suggestions to improve the support. Some of the topics in the guide for HCPs were their experience with care of C/ALHIV; how does the infection affect children and adolescents; issues related to ART and adherence; missed medications and maintenance of adherence to medications; support from the clinical team to C/ALHIV; issues that come us during disclosure of the status; changes in attitudes and behaviour of C/ALHIV after disclosure; concerns and issues related to disclosure; and challenges encountered in maintaining the treatment in children, adolescents, and their parents. Interviews were scheduled based on participant availability, held privately at ART centers, and audio-recorded with informed consent. Participants validated interview summaries at the end of each session. This helped the final analysis by confirming the accuracy of interpretations related to HIV disclosure experiences, helping in refining themes and ensuring that the findings closely reflected the lived experiences of C/ALHIV, caregivers, and HCPs. A note taker took notes on the person conducting the FGD or interview, site, duration, and participants in the interview.

Data management and analysis

The transcriptions and translations were done by the research team. Each recorded FGD and interview was given a unique alphanumeric identity number. We created separate folders for each FGD and interview. All the recorded interviews were transcribed in the original language of the interview (English, Hindi, or Marathi). The transcribed document also had the same identity number and was placed in the same digital folder. Each transcription was checked by another member of the research team. All the transcriptions (not in English) were then translated into English for further analysis. The translation was also checked by another team member (who was not primarily responsible for the translation). We also maintained a record of the following: team member who conducted the FGD/interview; team member who did the transcription; team member who did the translation; and team members who checked the transcriptions and translations. All the digital data (folder, transcription, and translation) had the same unique alphanumeric identity number.

We utilized thematic network analysis and manually summarized textual data into themes and their linkages in a thematic network [34-36]. Two researchers (PG and MD) coded each FGD and interview independently. These independent codes were discussed by both the researchers, and the decision on the final coding and theme generation was made by consensus. If required, input from the senior researcher (SA) was considered for the final codes. The basic themes were identified according to the specific phrases and concepts in the text by the researchers coding the data [36]. Within each coded theme, sub-themes were identified and labelled. After completing the analysis of one FGD/interview, the terms and labels for the codes were matched to ensure that the same term would be used for future analysis. If there was any discordance between the thematic coding of the data, it was resolved by consensus. The coding for each FGD/interview was discussed during a debriefing session with the senior researcher (SA), and, if, for some reason, consensus could not be achieved by both researchers coding the data, then input from the senior researcher was used to determine the theme and sub-theme. A list of all the themes and sub-themes was maintained for each FGD and interview, and this sheet was also given the same alphanumeric code as the original recorded interview. Both researchers also maintained a record of their thoughts during the coding process, and these were discussed during the debriefing sessions with the senior researcher. This process was followed for coding of each FGD and interview, and the next FGD/interview was coded only after completion of this process. After this, related themes were grouped into the organizing theme, and finally, the organizing themes were included in the overarching global theme [34]. For example, for the theme of "Apprehension of guardians and HCPs for HIV disclosure," the sub-themes were "Blame from the child" and "grief/shock experience by the C/ALHIV." These analyses helped us develop the non-hierarchical thematic network.

Ethics

The study received ethics approval from the Institutional Ethics Committee of Mumbai Districts AIDS Control Society, Mumbai, India (ID 002/2021, dated February 2, 2021). For participants over 17 years of age, informed written consent was obtained. For adolescents (aged 10-17 years), in addition to their assent for participation, consent was taken from their legal guardians for their participation.

## Results

Participant characteristics

In our four FGDs for ALHIV (two for female ALHIV and two for male ALHIV), 29 adolescents participated. The age of the ALHIV ranged from 14 to 19 years, and HIV status was disclosed to them at least six months before the time of interview. Eight adolescents (four males and four females) of ages between 16 and 19 years were interviewed to learn about their views and experiences of the disclosure process.

Four FGDs were conducted with primary caregivers of CLHIV and ALHIV (status undisclosed) and included 13 parents, three uncles/aunts, one brother, one grandparent, and one institutional caretaker. In addition, eight interviews were carried out with primary caregivers of CLHIV and ALHIV to explore their perceptions about disclosure. These interviews included five mothers and three fathers of CLHIV/ALHIV. Eight HCPs (two medical officers, two counselors, two care coordinators, and two outreach workers) at ART centers with more than two years of experience in the field were interviewed to learn their roles and experiences with the disclosure process.

HIV status disclosure: a challenging step in HIV care

**Figure 1:**
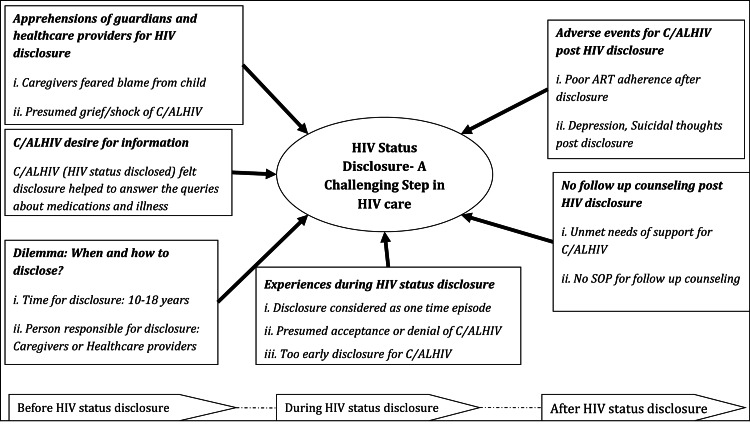
Non-hierarchical thematic network for "HIV Status Disclosure - A Challenging Step in HIV Care" in CLHIV and ALHIV on ART, Mumbai, India CLHIV: children living with HIV; ALHIV: adolescents living with HIV; SOP: standard operating procedure; ART: antiretroviral therapy Image credit: The figure was created by the authors using Microsoft Word (Microsoft Corp., Redmond, WA, USA).

The main finding of our qualitative analysis was that HIV status disclosure is a complex and challenging aspect of HIV care. We identified six key themes: (1) Apprehensions of guardians and HCPs for HIV status disclosure, (2) desire for information among ALHIV, (3) dilemma: when and how to disclose HIV status?, (4) experiences during disclosure, (5) adverse events for ALHIV post disclosure, and (6) absence of follow-up counseling post HIV disclosure. The themes are categorized into three groups as per the timeline of disclosure: pre-HIV status disclosure, HIV status disclosure, and post-HIV status disclosure. The thematic analysis in a non-hierarchical network is shown in Figure 1.

Pre-HIV Status Disclosure Apprehensions of Guardians and HCPs

Caregivers feared blame from children: Caregivers feared that the C/ALHIV, after knowing their HIV status, would blame them for their condition; hence, many caregivers preferred counselors to disclose the HIV status to their children.

"Some parents are scared to disclose as they fear how the child would react, if the child would remain on talking terms with them, if the child is going to hate them…" (HCP-1).

"We couldn’t gather the courage to inform her about her HIV status" (G3, Guardian of C/ALHIV-female).

"Disclosure depends on if the parents give consent. Some are not convinced and are afraid due to various reasons such as discrimination/phobia etc." (HCP-4).

Presumed grief/shock of C/ALHIV: Caregivers and HCPs worry that disclosing HIV status to children/adolescents could provoke strong emotions such as shock, denial, anger, or pain, affecting their school performance and adherence to treatment. However, C/ALHIV expressed that disclosure helped reduce their questions about medications and improved their understanding of their condition.

"I am concerned that if I tell counselors to inform her, she might get into shock… She might get frustrated. It scares me" (G3, Guardian of C/ALHIV-female).

"Some kids are already very angry about the fact that only they are on medications, and they question why others are not. They are already in denial. You have to go slow" (HCP-2).

Desire for Information Among C/ALHIV

C/ALHIV reported that it helped them understand their medications and illness better. They believed their same-aged peers should be informed about their HIV status, as having accurate information assists them in planning their lives and staying safe.

“Yes, he (any other C/ALHIV) should know what he has been consuming medicines for. Once, he gets old enough, he would understand how to be safe himself and how he can ensure others’ safety” (C/ALHIV-P1, FGD-2).

“They should be informed by parents. If they are not aware, they will feel that they are unnecessary taking medicines” (C/ALHIV-P4, FGD-3).

Dilemma: When and How to Start Disclosure?

Time for HIV status disclosure: The time for HIV status disclosure for C/ALHIV, proposed by HCPs, guardians, and C/ALHIV, varied between the ages of 10 and 18. The proposed age depended on the maturity of the C/ALHIV and the acceptance of the caregivers about the decision to disclose. This wide age range increased the dilemma around age-appropriate HIV status disclosure.

"Age is an important factor…In my observation, children below 10 years do not have any interest in knowing, and they are not mature enough to understand. But once they are in their teenage years, they are more curious to know what is happening with them" (HCP-1).

"We approach parents of adolescents whose age is more than 18 regarding disclosure" (HCP-3).

"Parents should be telling it to children aged beyond 9-10 years" (C/ALHIV-P6, FGD-2).

"I conduct age-appropriate disclosure at 12-13 years" (HCP-4).

Person responsible for HIV status disclosure: While HCPs and C/ALHIV preferred parents/caregivers to be the first person to initiate the disclosure, parents often felt doctors or counselors could handle it better.

"I keep thinking what to say, how to say, how he would react… Doctor Madam should tell them" (G1, Guardian of C/ALHIV-male).

"We insist that parents should be the first ones to tell the status to child. If they need help, then our counselors pitch in" (HCP-2).

"Parents should inform the children, otherwise they will keep on asking questions about the medicine" (C/ALHIV-P3, FGD-3).

Experiences During HIV Status Disclosure

Disclosure - one-time episode: Most HCPs and guardians felt that disclosure was a one-time activity, and all the apprehensions were related to informing C/ALHIV about HIV status. However, C/ALHIV reported that they did not get all the information about HIV status disclosure at one time, from parents or HCPs. They had to contact HCPs or other sources to get more information about HIV, ART, and its impact on life.

“Mother and father told me when I was in 8th std when we were at home. They told me that I have to take medicines for HIV… After coming to the center, after reading, I came to know… I searched on Google… I go to the doctor, but they haven’t informed me about my status” (C/ALHIV-5).

Presumed acceptance or denial of C/ALHIV: HCPs reported that most adolescents accept their HIV status disclosure during the initial meeting, but view acceptance as a one-time event rather than an ongoing process. Some adolescents experienced fear or anger following disclosure, and providers noted that they often had many questions about HIV during these sessions.

"In my observation, 99% of cases, these children were okay after knowing their status" (HCP-1).

"I have seen some adolescents, who come to know about their HIV status all of a sudden, without any prior hint, they become angry, sometimes aggressive… Some get emotional after disclosure" (HCP-3).

"Children have 100 questions about what the disease is, whether they have to take lifelong medicines" (HCP-5).

Too early disclosure for C/ALHIV: It was reported that HIV status was disclosed to some adolescents at a young age, and they did not fully understand it at the time. Later, some sought more detailed information, while others did not pursue additional clarification.

"When I was 11, a counselor explained to me everything about HIV. I did not understand it completely then…. When I turned 13, I walked up to the counselors and requested the information on HIV once again" (C/ALHIV-P1, FGD-4).

"I was 9 years old when I started ART. My mother told me… Later Doctor told me about it…. I was too young to comprehend that information" (C/ALHIV-3).

Adverse Events for C/ALHIV Post-HIV Status Disclosure

Poor ART adherence after HIV disclosure: HCPs reported that some adolescents took time to come to terms with their HIV status after disclosure, and many missed medication doses for a few weeks after knowing their status.

Depression, suicidal thoughts post-HIV disclosure: Some adolescents living with HIV develop depression, anxiety, and serious psychological issues such as suicidal thoughts, and require ongoing support to manage these challenges. This suggests that the disclosure process is not complete until the adolescent’s stress and anxiety related to their HIV status are effectively addressed and alleviated.

"Some children have suicidal tendencies after disclosure… Some of them don’t want to take medicines and are adamant" (HCP-4).

"Why me? Stress about transmission of the disease… I was a little upset and scared" (C/ALHIV-P3, FGD-4).

"My uncle had told me about my status when I was 13 years old. I got scared and went into depression hearing that" (C/ALHIV-P3, during FGD-1).

No Follow-Up Counseling Post-HIV Disclosure

Unmet needs of support for C/ALHIV: Many C/ALHIV continue to have queries post-HIV disclosure, even after, and are still seeking support for their anxiety and stress related to social life, work, and education while living with a chronic illness. The C/ALHIV were also worried about accidental disclosure of their HIV status at school, college, or the workplace.

"I am worried about transmitting it to my wife and kids, about getting rejected at the medical assessment level during the employment process" (C/ALHIV-P3, FGD-1).

No Standard Operating Procedure (SOP) for Follow-Up Counseling Post-HIV Disclosure

Absence of a protocol for routine follow-up counseling after HIV status disclosure was acknowledged. Some HCPs engaged with C/ALHIV to address their questions, while others concluded the disclosure process in a single session without further follow-up.

"As such, we do not have follow-up counselling" (HCP-1).

"They (counsellors) asked me if I understood the last session with them. Apart from that, no different treatment" (C/ALHIV-4).

Enablers and barriers for HIV status disclosure

The themes pertaining to enablers and barriers for the HIV disclosure process overlapped for C/ALHIV, caregivers, and HCPs, and therefore, they are described together (Table 2). Peer counselling, caregiver’s support, and individualized C/ALHIV counselling (in line with the interests of C/ALHIV) were identified as enablers, while gaps in knowledge of HCPs, incomplete age-appropriate counselling protocols, and sub-optimal documentation about disclosure were identified as barriers for the process of HIV status disclosure.

**Table 2:** Enablers and barriers to HIV status disclosure for children and adolescents living with HIV (C/ALHIV), Mumbai, India The first column lists the enablers and barriers, and the second column lists the quotes of the participants. HCP: healthcare provider

Enablers	
List of enablers	Participant quotes
Caregiver’s support	"He should be told about it by the family members. If he comes to know about it from outside, he might feel bad" (C/ALHIV-4).
Individualized C/ALHIV counselling (in line with interests of C/ALHIV)	"We need to understand what the children are interested in" (HCP-1).
Barriers	
List of barriers	Participant quotes
Non-availability of multiple platforms/formats for C/ALHIV for sharing their experiences or challenges	"No, I am not familiar with anyone else, because my timing is different; there is no one of my age at that time" (C/ALHIV-4).
Gap in knowledge related to HIV care in HCPs	"At some centers, medical officers and counselors have basic information, but in their case, revision training is needed (HCP-2) Counseling done by counselors is very bookish… counselors give them (C/ALHIV) strict schedule for timing of medication" (HCP-5).
Missing age-appropriate counseling protocol and tools	"I had no idea when and how disclosure counselling is done when I joined here" (HCP-2). "We look at the age of adolescent and if he/she has completed 18 years, then we go for disclosure if it hasn’t been done before. We generally do not make disclosure of HIV to children below the age of 18 years" (HCP-3). "Yes. I know about HIV... However, no one informed me in detail" (C/ALHIV-2).
Sub-optimal documentation about the disclosure in the ALHIV case file or treatment card	"I don’t write an elaborate note on disclosure counselling… just write it as counselling done" (HCP-3).
Lack of post-disclosure support	"Immediately after disclosure, there could be an irregularity in adherence for a month or so, it depends on level of maturity" (HCP-3). "Some adolescents when they come to know about their HIV status, they become angry, sometimes aggressive, blaming their parents for HIV status" (HCP-5).

Recommendations for improving HIV status disclosure

The participants also provided a few recommendations for improving the HIV status disclosure services, as mentioned in Table 3. The recommendations included training for HCPs, revised educational materials, age-appropriate counseling tools, and mandatory follow-up counseling sessions post-HIV status disclosure.

**Table 3:** Recommendations for improving HIV disclosure services for enablers and barriers to HIV status disclosure for children and adolescents living with HIV (C/ALHIV), Mumbai, India The first column lists the enablers and barriers, and the second column lists the quotes of the participants. FGD: focus group discussion, HCP: healthcare providers; IEC: information, education, and communication

Recommendation	Participant quotes
1. Training for HIV disclosure counseling for HCPs	"We should have guidelines/training on the quality of counseling. what are the new strategies in counseling to improve adherence?" (HCP-3).
2. Frequently asked questions (IEC material) could be developed and distributed to ALHIVs	"There should be some ready answers for their questions regarding the routine blood investigations they are advised to undergo" (HCP-5).
3. Counseling tools should be developed age-wise	"You cannot use the same model of counseling for the child who had come to us at the age of 2 years, and now he is 4 years old. The child has grown up, so have his needs" (HCP-5).
4. Follow-up disclosure counseling for everyone at a particular age, as the C/ALHIV who know about their status at an early age may not have understood in detail at that age	"When I was in 7th standard, my mother had told me about it. I was too young. I didn’t understand much about it then" (C/ALHIV-P4, FGD-2).

## Discussion

This study explored the perspectives of C/ALHIV, their guardians, and HCPs on HIV disclosure. It highlights the complex and sensitive nature of informing children and adolescents about their HIV status.

In the context of the age of disclosing HIV status to adolescents, our participants (guardians, healthcare workers, and C/ALHIVs) showed consensus that the age of 10-18 is appropriate for the disclosure process. Other studies, however, have given other recommendations. For instance, in Kiwanuka's study (Uganda), caregivers suggested an age of 12-15 [12], while Gyamfi et al. (Ghana) reported that many caregivers considered children under 13 years too immature to understand their illness [37]. In Baker et al.'s study (Peru), children themselves suggested starting the process much earlier, between the ages of six and seven [38]. Das et al. conducted a study in West Bengal, India, where some caregivers suggested delaying disclosure until the child reached a "marriageable age" [19]. WHO guidelines recommend initiating the HIV disclosure process at age six and completing it before a child turns 12, and the Indian national guidelines recommend initiating the process at four to six years and completing it by 12-14 years [21,30]. However, the implementation of the HIV disclosure process varied among ART centres in the city, with pediatric ART centres practicing age-appropriate disclosure using locally developed audiovisual (A-V) aids, while other ART centres disclosed around 17-18 years, after discussion with caregivers. Research from other countries has also documented that C/ALHIV are often informed of their status much later in life, typically between ages 17 and 18 [10,14]. The cultural beliefs that children are too immature to cope, fears that they will become depressed or lose hope, and concerns regarding social discrimination have been important factors delaying the disclosure. In our study, fear about accidental disclosure in the community through C/ALHIV and feelings of guilt among the parents delayed the disclosure.

Stakeholders also disagreed on who should disclose the status. While our study found that both HCPs and C/ALHIVs believed parents/caregivers should initiate disclosure, caregivers often felt that doctors or counselors were better suited for this task, fearing that children might blame them for the infection or react with shock, denial, anger, or sadness, potentially affecting academics or ART adherence. This concern is consistent throughout the numerous studies conducted on disclosure [10,19,39,40]. Das et al. reported that caregivers felt HCPs were best equipped to disclose, citing their superior knowledge and ability to explain the disease effectively [19]. In contrast to this, Madiba [10] found a lack of consensus among healthcare workers themselves, where some believed caregivers should lead the process, while others felt that healthcare workers should handle disclosure in specific scenarios, such as when a caregiver refuses to, provides false information, or requests assistance. A collaborative approach was also suggested, where disclosure is a joint responsibility [10]. Regardless of who initiates the conversation, it is agreed that post-disclosure follow-up for both the adolescent and the caregiver is a critical element for ensuring treatment and adherence [40].

Our findings also showed a mismatch in how disclosure is perceived: caregivers and providers often saw it as a one-time event, whereas C/ALHIV described it as an incomplete process, requiring them to seek additional information later. Bingaman et al. (USA) reported that most caregivers considered disclosure as a process that unfolds over time [41]. These findings support the WHO recommendation of an incremental, developmentally appropriate approach. We observed that while most C/ALHIV accepted disclosure during the meeting, children often reacted with fear or anger and raised multiple queries regarding their illness. Studies report mixed responses during disclosure, with the majority displaying strong negative emotions initially. However, reassurance and continued communication improved outcomes for many [9,42]. Kiwanuka et al. (Uganda) reported that caregivers were concerned about disclosure leading to self-harm and suicide [12]. However, the HCPs in our study were not aware of the need for emotional and psychological support for the adolescents during the post-disclosure period. These findings highlight that disclosure is not only an emotionally charged event but also a process requiring sustained support.

Notably, we observed that participants from the pediatric ART center showed greater awareness and better disclosure practices than those from other ART centers. This difference may be due to more effective context-specific training and sensitization of HCPs at the pediatric ART center, and these findings may not apply to other ART centers. The documentation of disclosure counselling on the treatment records for follow-up services was lacking at all the centers. Even after disclosure, most C/ALHIV continued to struggle with anxiety related to social life, education, work, and fear of unintended disclosure in schools or workplaces. This aligns with global evidence highlighting long-term psychological impacts and the need for structured disclosure models. Many such models have been proven to be effective [43]. HCPs in our study acknowledged their limited skills and lack of protocols and guidelines for disclosure and follow-up counselling. These findings underscore the urgent need to develop a comprehensive protocol specific to the cultural and healthcare needs of the community, alongside capacity-building of HCPs to provide safe, staged, and supportive disclosure involving the parents and caregivers in the process.

Limitations

The study had limitations. Since this was a qualitative study with purposive sampling, there is limited generalizability of the results. Other authors have used quantitative methods, and these can be explored in further studies [44,45]. All the FGDs and in-depth interviews were based in Mumbai. HIV control and treatment programme activities have been implemented in the city since the beginning. Thus, some issues related to acceptance of HIV, stigma, and discrimination may be different from other cities where the HIV control and management programmes were started later. Thus, specific cultural and local issues from other parts of the country may not have been captured in this study. Though we interviewed HCPs, we did not include government stakeholders in this study. We propose to discuss and share these findings with the government stakeholders at the local and national levels. Most of these participants were recruited from the ART centres and were in care for some time. Thus, some responses and discussions may have been influenced by social desirability bias in these settings [46]. They may not be comfortable saying some issues related to ART care and disclosure. Furthermore, as reported earlier, even though a preliminary review of the recorded data of each FGD and interview was conducted to identify initial themes before conducting the next FGD/interview, a formal analysis for thematic saturation was not done [32,33]. It is likely that this may have resulted in some themes not being identified. Thus, the results from the study should be interpreted within the context of these limitations.

## Conclusions

Based on our results, we underscore the importance of strengthening existing approaches to HIV disclosure for children and adolescents receiving care at ART centers. Although national guidelines exist, they do not comprehensively address the nuanced challenges surrounding staged, age-appropriate disclosure in varied local community contexts. We advocate the development of comprehensive national guidance that incorporates cultural considerations and practical family-centered strategies. Strengthening the capacity of HCPs - through training in child psychology, effective communication, and counselling skills - is critical to facilitating this process. Collaborative disclosure models that involve both caregivers and HCPs should be promoted to create a more supportive environment for children. Disclosure should also be viewed as an ongoing process rather than a one-time event, supported by structured follow-up mechanisms that provide ongoing psychosocial care and promote high-quality health services for adolescents living with HIV. The structured psychosocial support interventions with proper documentation need to be integrated within the disclosure process so that the C/ALHIV receive continuous emotional support to cope with any evolving distress situation due to disclosure.
